# Factors Associated With Job Precariousness and Material Deprivation, and Their Association With Mental Health During the COVID‐19 Pandemic

**DOI:** 10.1111/sltb.70132

**Published:** 2026-07-28

**Authors:** Sheila Lopez‐Romeo, Pere Castellvi, Isabel Feria‐Raposo, Andrea Miranda‐Mendizabal, Silvia Recoder, Marc Casajuana‐Closas, Susana Subira‐Alvarez, Carlos G. Forero

**Affiliations:** ^1^ Department of Clinical and Health Psychology Universitat Autonoma de Barcelona (UAB) Bellaterra Spain; ^2^ Fundacio Hospitalaries Sant Boi Sant Boi de Llobregat Spain; ^3^ Department of Medicine, School of Medicine and Health Sciences Universitat Internacional de Catalunya (UIC) Sant Cugat del Vallès Spain; ^4^ Nursing and Healthcare Research Unit (Investén‐ISCIII) Instituto de Salud Carlos III Madrid Spain; ^5^ FIDMAG Germanes Hospitalaries Research Foundation Barcelona Spain; ^6^ Mental Health Networking Biomedical Research Centre (CIBERSAM) Madrid Spain; ^7^ Sant Joan de Déu Research Institute Esplugues de Llobregat Spain; ^8^ Department of Basic Sciences Universitat Internacional de Catalunya (UIC) Sant Cugat del Vallès Spain; ^9^ Institut Universitari de Investigació en Atenció Primaria Jordi Gol (IDIAP Jordi Gol) Barcelona Spain

**Keywords:** COVID‐19, mental health, poverty, precarious employment, resilience, suicide

## Abstract

**Introduction:**

COVID‐19 severely disrupted labor markets and living conditions, intensifying job precariousness and material deprivation. We examined whether pre‐pandemic mental health conditions predicted socio‐economic adversity during the first pandemic year and whether resilience buffered changes in mental health.

**Methods:**

We analyzed a nationally representative longitudinal cohort of Spanish adults assessed in two assessments: November 2019 (T0, pre‐pandemic) and November–December 2020 (T1). Major depressive episode (MDE), generalized anxiety disorder (GAD), and suicidal thoughts and behaviors (STB) were assessed at T0; job precariousness and material deprivation were measured at T1. Weighted multivariable regression models tested (i) associations between T0 mental health and T1 socio‐economic adversity and (ii) associations between T1 adversity and changes in mental health symptoms, including resilience interactions.

**Results:**

Pre‐pandemic GAD and STB were associated with higher odds of job precariousness during the pandemic, whereas MDE was not significant after adjustment. Job precariousness was associated with small reductions in depressive, anxiety, and STB symptoms, while material deprivation was associated with increased depressive symptoms; higher resilience attenuated the deprivation–depression association.

**Conclusions:**

Pre‐existing anxiety and suicide‐related symptoms may identify individuals more likely to report job precariousness during socio‐economic shocks. Preventing material deprivation and strengthening resilience may help reduce downstream depressive burden during crises.

## 1 Introduction

The COVID‐19 pandemic constituted a global public health emergency with far‐reaching social and economic consequences that extended well beyond the direct effects of infection and mortality. Measures implemented to contain viral transmission, such as lockdowns, mobility restrictions, and business closures, produced abrupt disruptions in labor markets and household economies, generating widespread employment instability and financial insecurity (Bambra et al. 2020; Blundell et al. 2020). These disruptions occurred within a context of pre‐existing social inequalities, which the pandemic both exposed and intensified. Beyond these structural disruptions, the pandemic also amplified vulnerability in specific population subgroups, with psychosocial stressors and restrictions being associated with poorer wellbeing and quality of life. Young people, in particular, have been highlighted as especially susceptible to stress‐related psychosocial consequences during the COVID‐19 period (Amerio et al. 2020).

Job precariousness and material deprivation emerged as central dimensions of this socio‐economic shock. Job precariousness encompasses employment arrangements characterized by instability, low‐income security, limited social protection, and reduced bargaining power (Benach et al. 2014; Kreshpaj et al. 2020). Material deprivation, in turn, refers to the enforced inability to afford goods, services, and living conditions considered necessary for a minimally acceptable standard of living (Atkinson and Marlier 2010). Both conditions have been consistently associated with adverse mental health outcomes, including depressive and anxiety disorders, psychological distress, and suicidal thoughts and behaviors (STB) (Kim and Von Knesebeck 2015; Min et al. 2014).

During periods of economic crisis, the relationship between socio‐economic adversity and mental health appears to be particularly pronounced. Evidence from previous recessions, including the 2008 global financial crisis, indicates that unemployment, job insecurity, and financial strain are associated with increased rates of depression, anxiety, and suicide (Karanikolos et al. 2013; Reeves et al. 2015; Stuckler et al. 2009). The COVID‐19 pandemic shares key features with these crises, while also presenting distinctive characteristics, such as its sudden onset, global scale, and the normalization of employment instability across broad segments of the population.

This contextual shift raises important questions regarding the psychological meaning of job precariousness during the pandemic. When employment instability becomes widespread and collectively experienced, its subjective interpretation may differ from that observed under “normal” economic conditions. Some authors have suggested that, in such contexts, precarious employment may be perceived less as an individual failure and more as a shared societal condition, potentially altering its association with mental health outcomes (Pfefferbaum and North 2020). Empirical evidence examining these dynamics during the COVID‐19 pandemic, however, remains limited.

Another critical but underexplored dimension concerns the role of pre‐existing mental health conditions in shaping exposure to socio‐economic adversity. Individuals with depression, anxiety disorders, or STB may be particularly vulnerable to adverse employment trajectories due to factors such as reduced work capacity, stigma, or cumulative disadvantage (Benach et al. 2014; Vives et al. 2013). Longitudinal evidence assessing whether mental health conditions present before a large‐scale socio‐economic shock increase the likelihood of subsequent job precariousness or material deprivation is scarce, particularly in population‐based samples.

Furthermore, socio‐economic adversity does not affect all individuals uniformly. Resilience, commonly defined as the capacity to adapt positively in the face of stress and adversity, has been identified as a key protective factor in mental health research (Bonanno et al. 2011; Connor and Davidson 2003). Higher levels of resilience have been associated with lower depressive symptomatology and reduced psychological distress among individuals exposed to economic hardship and other stressors (Jeamjitvibool et al. 2022). However, the extent to which resilience moderates the relationship between job precariousness, material deprivation, and mental health outcomes during population‐level crises such as the COVID‐19 pandemic remains insufficiently understood.

Using data from a nationally representative longitudinal cohort of Spanish adults assessed before and during the first year of the COVID‐19 pandemic, the present study addresses these gaps by adopting an analytical perspective focused on socio‐economic vulnerability and mental health. Specifically, this study aims to: (1) examine whether pre‐pandemic mental health conditions; major depressive episode (MDE), generalized anxiety disorder (GAD), and STB, are associated with an increased risk of job precariousness and material deprivation during the pandemic; (2) assess the associations between exposure to job precariousness and material deprivation and changes in mental health symptoms over time and their interaction with age; and (3) evaluate whether resilience moderates the impact of these socio‐economic stressors on mental health outcomes. Based on prior evidence linking socio‐economic adversity to mental health and suicide‐related outcomes, we hypothesized that: (H_1_) pre‐pandemic mental health conditions (MDE, GAD, and STB) would be associated with a higher likelihood of experiencing job precariousness and material deprivation during the COVID‐19 pandemic; (H_2_) exposure to job precariousness and material deprivation during the pandemic would be associated with worsening mental health outcomes over time (i.e., increased depressive symptoms, anxiety symptoms, and STB severity), and this association would be different across age groups; and (H_3_) resilience would attenuate the adverse associations between these socio‐economic stressors and changes in mental health outcomes.

By clarifying the temporal relationships between mental health, socio‐economic vulnerability, and resilience, this study seeks to contribute to a more nuanced understanding of suicide‐related risk and protective mechanisms during large‐scale socio‐economic crises. Such evidence is essential for informing public health and social policies aimed at mitigating not only economic hardship but also its mental health and suicide‐related consequences among vulnerable populations.

## 2 Materials and Methods

### 2.1 Study Design and Data Source

This study used data from a population‐based longitudinal cohort designed to assess health and social outcomes in the Spanish adult population. Participants completed two online assessments: a baseline survey conducted prior to the COVID‐19 pandemic (November 2019) and a follow‐up survey administered approximately 1 year later, during the first year of the pandemic (November–December 2020). The baseline assessment was carried out before the onset of SARS‐CoV‐2 transmission worldwide, allowing for the prospective evaluation of mental health conditions prior to the socio‐economic disruptions associated with the pandemic.

The study protocol was approved by the relevant institutional ethics committee (Reg. Number: MED‐2020‐02), and all participants provided informed consent prior to participation. Data were anonymized before analysis. Information on the rationale and methods of the BIOVAL‐D‐COVID‐19 project has been published elsewhere (Miranda‐Mendizabal et al. 2022).

### 2.2 Study Population and Sampling

The baseline sample consisted of a nationally representative cohort of adults aged 18 years and older, selected to reflect the Spanish population in terms of age, sex, geographic distribution, and socioeconomic status. Participants were recruited from the Kantar Spain Access Panel, a closed, proprietary probability‐based online panel compliant with ISO 20252:2019 standards. Panel members (≥ 18 years) were recruited from private households through random sampling of phone numbers (landlines and mobiles) using a stratified multistage approach (municipalities stratified by Spain's 17 Autonomous Communities; census tracts selected by municipality size; individuals randomly selected within tracts). During panel recruitment, brief interviews were used to calibrate panel composition to the Spanish census distribution in terms of sex, age, geographic region, and key sociodemographic characteristics. For the present study, a study sample was invited from within the panel (selected without replacement) and administered an online survey at baseline (T0) and follow‐up (T1). Although the underlying panel is probability‐based, participation in a given study depends on invitation and response within the panel; therefore, we applied post‐stratification survey weights to approximate population margins. To minimize non‐response, invitees received up to three follow‐up contacts. At follow‐up, we additionally applied inverse probability‐of‐censoring weights to mitigate differential attrition between T0 and T1.

At baseline (T0), 2005 individuals completed the survey. Some instruments were administered to planned subsamples at baseline; therefore, analytic sample sizes vary across outcomes and models (model‐specific Ns are reported in the tables). At follow‐up (T1), all baseline participants were invited to participate again. All participants provided informed consent prior to participation; respondents who did not complete the follow‐up questionnaire were not included in longitudinal analyses.

A total of 922 participants completed both assessments and were included in the present analyses. Because some baseline instruments were administered to subsamples and because of item non‐response, analytic sample sizes differed across outcomes and models.

In particular, at baseline the GAD‐7 was administered to all participants screening positive for depression (CIDI) and to a randomized 40% of those screening negative; at follow‐up, the GAD‐7 was administered to the full sample (and WHODAS 2.0, where applicable). Therefore, analyses involving GAD symptom changes from T0 to T1 were restricted to participants who completed the GAD‐7 at both assessments (*n* = 313), and model‐specific sample sizes are reported in the tables. Weighting procedures have been described elsewhere (Castellvi Obiols et al. 2023). In addition, during the first pandemic year in Spain, job‐retention and temporary layoff arrangements (ERTE‐type furlough schemes) were widely implemented; however, individual furlough/ERTE status and leave‐of‐absence arrangements (e.g., work leave) were not specifically captured in our survey. Employment status was therefore modeled using broad categories (e.g., employed, unemployed, retired, student), and we could not adjust for furlough/leave status at the individual level.

### 2.3 Mental Health Measures

#### 2.3.1 Major Depressive Episode (MDE)

Major depressive episode was assessed using the Spanish version of the Composite International Diagnostic Interview (CIDI), version 3.0 (Haro et al. 2006; Kessler and Üstün 2004). The instrument includes a screening section followed by a diagnostic module that evaluates the presence of depressive symptoms lasting at least 2 weeks. Diagnostic criteria were met when participants endorsed five or more symptoms accompanied by significant functional impairment, operationalized as a disability score greater than 50 on the World Health Organization Disability Assessment Schedule (WHODAS) (Luciano et al. 2010; Vázquez‐Barquero et al. 2000).

#### 2.3.2 Generalized Anxiety Disorder

**FIGURE 1:**
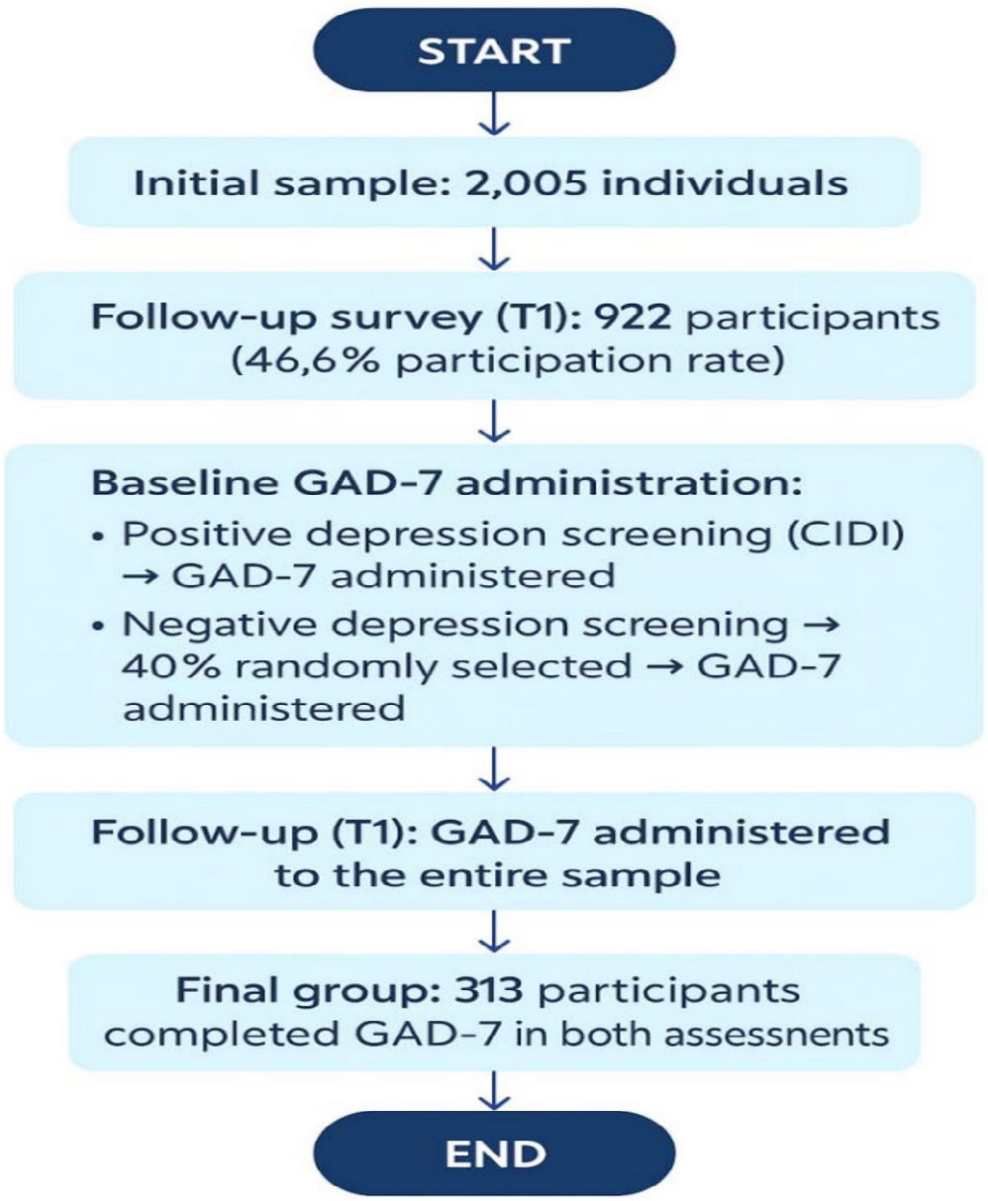
Participant flow and GAD‐7 administration process. At baseline, GAD‐7 was administered to all participants screening positive for depression (CIDI) and to a random 40% of those screening negative; at follow‐up, it was administered to the full sample. Overall, 313 participants completed GAD‐7 at both assessments.

GAD was assessed using the GAD–7 scale (García‐Campayo et al. 2010), a validated 7‐item self‐report measure of anxiety symptom severity over the past 2 weeks (total score range 0–21). Items are rated on a four‐point Likert scale, with higher scores indicating greater symptom severity. The Spanish version has shown excellent internal consistency (Cronbach's *α* = 0.936) and good test–retest reliability (García‐Campayo et al. 2010). A GAD‐7 score of ≥ 10 indicates at least moderate anxiety symptom severity and is commonly used as a screening threshold for probable GAD in community samples. In this study, we classified probable GAD as a score of ≥ 10 combined with significant functional impairment. The GAD‐7 administration process is shown in Figure 1.

#### 2.3.3 Suicidal Thoughts and Behaviors

STB, a commonly used umbrella term in the suicide literature (Nock 2010), were assessed using selected items from the CIDI covering passive suicidal ideation, active ideation, suicide planning, and suicide attempts. Participants were classified as reporting STB if they endorsed any of these items. In addition, a continuous suicide risk score was constructed by weighting different suicidal behaviors according to severity.

Mental health conditions were analyzed as both dichotomous and continuous variables, depending on the specific research objective.

In the present analyses, the primary mental health conditions were MDE and GAD, and we additionally examined STB; other psychiatric disorders were not analyzed as primary outcomes in this manuscript.

### 2.4 Job Precariousness

Job precariousness was assessed at follow‐up (T1) using indicators derived from the Employment Precarity Index (Bodin et al. 2020; Kreshpaj et al. 2020; Precarious Work Research 2025). This multidimensional construct captures employment instability, income insecurity, limited labor rights, fear of job loss or retaliation, and irregular working conditions. Accordingly, the measure reflects both objective employment conditions and subjective perceptions of employment insecurity, including fear of job loss or retaliation. Participants were classified as experiencing job precariousness if they met at least one of the predefined criteria.

Participants were classified as experiencing job precariousness if they met at least one of the predefined criteria. Detailed operational definitions and item‐level criteria for job precariousness and material deprivation are provided in the Methods S1 and Table S1. The index was applied to individuals who were employed, studying, or had experienced job loss during the months preceding the follow‐up assessment. Operationally, job precariousness was coded only for labor‐force active participants; those not in the labor force (e.g., retired) were coded as ineligible for job precariousness assessment and were excluded from analyses where job precariousness was the dependent variable. Accordingly, analyses in which job precariousness was the dependent variable were estimated in the labor‐force active sub‐sample, and model‐specific sample sizes are reported in Table 2.

### 2.5 Material Deprivation

Material deprivation was assessed at follow‐up using Eurostat‐aligned indicators of the inability to afford essential goods and living conditions. Material deprivation was assessed at follow‐up using indicators aligned with Eurostat definitions. Participants reported whether they were able to afford essential goods and living conditions, including adequate home heating, a protein‐rich diet, the capacity to manage unexpected expenses, and the ability to take an annual vacation. Material deprivation was defined as the inability to afford at least three of the four assessed items (Atkinson and Marlier 2010).

Further operational definitions and item‐level criteria are provided in Methods S1 and Table S1.

### 2.6 Covariates

Potential confounding variables included age, sex, civil status, employment status, physical comorbidities, body mass index, and disability. Employment status was self‐reported at baseline (T0) using predefined categories (e.g., employed, unemployed, student, retired, sick leave) and was included as a categorical covariate in adjusted models. Disability/functional impairment was assessed using the 12‐item World Health Organization Disability Assessment Schedule 2.0 (WHODAS 2.0), a generic measure of functioning across major life domains, with higher scores indicating greater disability (Vázquez‐Barquero et al. 2000). The 12‐item self‐report WHODAS 2.0 has shown high internal consistency (Cronbach's *α* = 0.83–0.92) and good 2‐week test–retest reliability (ICC = 0.83) in online administration (Axelsson et al. 2017). Physical comorbidities were identified through self‐reported physician diagnoses of chronic conditions using the European Study of the Epidemiology of Mental Disorders/Mental Health Disability (ESEMeD) (Alonso et al. 2004). COVID‐19 exposure variables, including testing status, symptoms, hospitalization, and COVID‐19–related stressors, were also considered in adjusted models. Unless otherwise specified, sociodemographic and health‐related covariates were measured at baseline (T0). COVID‐19 exposure variables and pandemic‐related stressors were measured at follow‐up (T1).

### 2.7 Resilience

Resilience was measured using the 10‐item Connor–Davidson Resilience Scale (CD‐RISC) (Notario‐Pacheco et al. 2011). Items were rated on a five‐point Likert scale, with higher scores indicating greater resilience (Connor and Davidson 2003). The Spanish 10‐item CD‐RISC has demonstrated good internal consistency (Cronbach's *α* = 0.85).

### 2.8 Statistical Analysis

Descriptive analyses were conducted using means and standard deviations for continuous variables and frequencies and percentages for categorical variables. Associations between pre‐pandemic mental health conditions and job precariousness or material deprivation were examined using logistic regression models. Changes in mental health symptoms over time were analyzed using linear regression models.

To explore potential differences across age groups, initial analyses were conducted using age‐stratified models (18–29, 30–65, and ≥ 66 years). Within each age group, regression models were estimated to assess the association between job precariousness and changes in mental health outcomes. These stratified analyses provided an exploratory assessment of potential heterogeneity across age groups.

However, given the limitations inherent to stratified analyses, particularly reduced statistical power and potential instability in smaller subgroups, we further examined age‐related differences by formally testing interaction effects. Specifically, interaction terms between age group and job precariousness and material deprivation were included in the regression models, using the 30–65 years group as the reference category.

All models were progressively adjusted for potential confounders. Interaction terms were included to assess the moderating effect of resilience on the relationship between socio‐economic stressors and mental health outcomes. Survey weights and inverse probability‐of‐censoring weights were applied in all analyses to account for sampling calibration and attrition. Statistical significance was set at *p* < 0.05. Analyses were conducted using R and SPSS (version 20.0).

To assess potential attrition bias, baseline characteristics of participants who completed both the baseline and follow‐up assessments were compared with those who participated only at baseline. Differences between groups were examined using chi‐square tests for categorical variables and *t*‐test for continuous variables. Inverse probability‐of‐censoring weights were derived from the estimated probability of participation at follow‐up based on baseline characteristics.

The present analyses were not formally pre‐registered; the study protocol received ethics approval and project methods have been described elsewhere (Miranda‐Mendizabal et al. 2022).

All regression analyses were conducted using complete‐case data for the variables required by each model; consequently, sample sizes vary across models. The main longitudinal cohort included 922 participants; analyses involving longitudinal GAD‐7 outcomes were restricted to the subset with GAD‐7 data at both assessments (*n* = 313). For transparency, the N included in each regression model is provided in Tables 2 and 3.

## 3 Results

### 3.1 Sample Characteristics

The follow‐up cohort comprised 922 participants who completed both the pre‐pandemic baseline assessment and the follow‐up survey during the first year of the COVID‐19 pandemic. Since several measures were collected in subsamples (notably baseline GAD‐7) and due to missing data, analytic sample sizes vary across outcomes and models (see Tables 2 and 3 for model‐specific Ns and Table 1 for missingness).

**TABLE 1:** Baseline characteristics of participants retained at follow‐up (T1): absolute numbers and weighted proportions.

Variables	Total *N* = 922 *N* (%)
*At baseline*	
Mental health condition	
MDE	53 (6.1)
GAD (Missing = 609)	36 (9.5)
STB (Missing = 18)	112 (12.4)
Passive ideation	107 (11.6)
Active ideation	26 (2.8)
Plan	13 (1.4)
Attempt	6 (0.7)
*Socio‐demographic variables*	
Age	
Mean (SD)	49.9 (15.7)
18–25	41 (4.5)
26–40	146 (15.8)
41–65	534 (57.9)
66 or older	201 (21.8)
Gender	
Men	450 (48.8)
Women	472 (51.2)
Civil status	
Single	244 (26.5)
Married/Living with a couple	544 (58.9)
Separated	15 (1.7)
Divorced	64 (6.9)
Widowed	55 (6.0)
Employment status	
Employed	481 (52.2)
Sick leave (> 3 months)	12 (1.3)
Unemployed	70 (7.6)
Housework	59 (6.4)
Student	22 (2.4)
Student and employed	16 (1.8)
Disability allowance	26 (2.8)
Retired	236 (25.5)
*Job precariousness*	
Score (0–5), mean (SD)	1.25 (1.34)
0	386 (42.9%)
1	162 (17.5%)
2	179 (20.2%)
3	112 (12.0%)
4	53 (6.3%)
5	13 (1.1%)
Material deprivation	
Score (0–4), mean (SD)	0.48 (0.92)
0	871 (73.5%)
1	156 (12.1%)
2	114 (8.9%)
3	51 (4.3%)
4	12 (1.3%)
*General health status*	
Self‐perceived general health	
Excellent	50 (5.4)
Very good	180 (19.5)
Good	536 (58.2)
Fair	140 (15.2)
Poor	16 (1.7)
Smoking status	
Non smoker	805 (87.3)
Smoker	117 (12.7)
BMI (Missing = 3)	
Underweight	18 (2.0)
Normal	415 (45.2)
Overweight	359 (39.0)
Moderately obese	104 (11.3)
Severely obese	19 (2.1)
Very severely obese	4 (0.4)
*Comorbidity*	
Chronic Diseases	
AIDS or HIV infection	2 (0.2)
Allergy	222 (24.1)
Back pain	188 (20.4)
Cancer	44 (4.7)
Cardio‐vascular	33 (3.6)
Diabetes	51 (5.5)
Fibromyalgia	27 (3.0)
Gastrointestinal	26 (2.8)
Headache	135 (14.6)
Hypertension	177 (19.2)
Neurological	9 (1.0)
Osteoarthritis	147 (15.9)
Respiratory	32 (3.5)
Stroke	15 (1.6)
Thyroid	59 (6.3)
None of the above	305 (33)
Number of chronic diseases	
0	326 (35.5)
1	275 (29.8)
2	172 (18.6)
3	82 (8.9)
> 3	67 (7.2)
WHODAS −12 (Missing = 455)	Median (Q1–Q3) 17.0 (13.0–24.0)
*At 12‐month follow‐up*	
COVID‐19 contagion	
No COVID test done and no symptoms	556 (60.3)
COVID‐19 symptoms without COVID test done	25 (2.7)
COVID‐19 test done and negative result	311 (33.8)
COVID‐19 test done and positive result	30 (3.2)

*Note:* Percentages are weighted (inverse probability‐of‐censoring weights and post‐stratification). Mean (SD) values for job precariousness and material deprivation scores were computed from weighted distributions. Mental health indicators were derived from validated screening instruments using standard cut‐offs with impairment criteria (see Section 2 and Methods S1 for operational definitions). Job precariousness was operationalized as the count of five criteria (0–5) and analyzed both as a count and as exposed (≥ 1 criterion). Material deprivation was operationalized as the count of four Eurostat‐aligned items (0–4) and analyzed both as a count and as deprived (≥ 3 items). Detailed operational definitions and item‐level criteria are provided in Methods S1 and Table S1.

Abbreviations: AIDS = acquired immunodeficiency syndrome, BMI = body mass index, COVID‐19 = coronavirus disease 2019, GAD = generalized anxiety disorder, HIV = human immunodeficiency virus, MDE = major depressive episode, STB = suicidal thoughts and behaviors, WHODAS = World Health Organization Disability Assessment Schedule.

Women represented 51.2% of the sample, and the mean age was 49.9 years (SD = 15.7). Most participants were married or living with a partner (58.9%), and slightly more than half were employed or combining employment with studies at follow‐up (54.0%). A substantial proportion of participants were retired (25.5%), while a small minority were on sick leave (1.3%).

Because job precariousness was defined for participants who were labor‐force active (employed, studying, or recently jobless), analyses involving job precariousness were conducted in a subset of the follow‐up cohort, whereas material deprivation analyses retained a broader sample; model‐specific Ns are provided in Tables 2 and 3.

**TABLE 2:** Association between mental health conditions before the COVID‐19 pandemic and job precariousness and material deprivation during the COVID‐19 pandemic.

Mental health condition before the pandemic	Job precariousness		Material deprivation	
	Model 1: bivariate	Model 2: multivariate	Model 1: bivariate	Model 2: multivariate
	*N* OR (95% CI)	*N* aOR (95% CI)	*N* OR (95% CI)	*N* aOR (95% CI)
MDE	*n* = 627 2.68 (1.44–4.98)**	*n* = 625 1.36 (0.53–3.50)	*n* = 856 1.98 (1.10–3.55)*	*n* = 852 0.63 (0.28–1.39)
GAD	*n* = 204 1.94 (0.94–3.98)	*n* = 203 3.24 (1.15–9.14)*	*n* = 285 2.12 (1.04–4.34)*	*n* = 282 1.67 (0.70–4.01)
STB	*n* = 620 2.74 (1.81–4.14)**	*n* = 618 3.01 (1.55–5.84)**	*n* = 844 2.06 (1.33–3.18)**	*n* = 840 1.14 (0.64–2.02)

*Note:* Model 2 adjusts for age, gender, civil status, self‐perceived physical health, self‐perceived mental health, smoking status, BMI, and number of chronic diseases. GAD‐7 at baseline was administered to all participants screening positive for depression and to a random 40% of those screening negative; at follow‐up it was administered to the full sample. Operational definitions for mental health indicators, job precariousness, and material deprivation are provided in the Section 2 and Methods S1. *p < 0.05; **p < 0.01.

**TABLE 3:** Association between job precariousness and material deprivation during the COVID‐19 pandemic and changes in the number of mental health symptoms before and during the pandemic, including resilience as a moderator variable.

	Change in the number of symptoms		
	MDE	GAD	STB
	*N* = 627 *β* (95% CI)	*N* = 204 *β* (95% CI)	*N* = 620 *β* (95% CI)
Job precariousness	−0.146 (−0.403 to 0.110)	−0.176 (−0.334 to −0.017)*	−0.334 (−0.581 to −0.087)*
Plus potentially confounding variables	−0.598 (−0.919 to −0.277)**	−0.355 (−0.528 to −0.183)*	−0.577 (−0.882 to −0.272)**
Resilience iteration	−0.003 (−0.040 to 0.035)	−0.037 (−0.074 to −0.001)*	−0.007 (−0.043 to 0.029)
Resilience iteration plus potentially confounding variables	−0.002 (−0.035 to 0.038)	−0.035 (−0.074 to 0.003)	0.004 (−0.031 to 0.039)

	*N* = 856 *β* (95% CI)	*N* = 285 *β* (95% CI)	*N* = 844 *β* (95% CI)
Material deprivation	0.497 (0.256 to 0.738)**	0.029 (−0.093 to 0.150)	0.096 (−0.155 to 0.348)
Plus potentially confounding variables	0.496 (0.253 to 0.740)**	0.066 (−0.064 to 0.197)	0.140 (−0.112 to 0.393)
Resilience iteration	−0.075 (−0.107 to −0.042)**	−0.016 (−0.034 to 0.002)	0.004 (−0.030 to 0.038)
Resilience iteration plus potentially confounding variables	−0.078 (−0.111 to −0.046)**	−0.016 (−0.034 to 0.002)	−0.005 (−0.038 to 0.029)

*Note:* Change scores were computed as T1 minus T0 symptom counts (continuous). Models adjust for age, gender, civil status, self‐perceived physical health, self‐perceived mental health, smoking status, BMI, and the number of chronic diseases. Estimates were weighted using post‐stratification and inverse probability‐of‐censoring weights. Ns correspond to available complete‐case observations and therefore vary across outcomes and models. Details on GAD‐7 administration and operational definitions of job precariousness and material deprivation are provided in the Section 2 and Methods S1. *p < 0.05; **p < 0.01.

A sensitivity analysis was conducted to examine potential attrition bias by comparing baseline characteristics of participants who completed both survey assessments with those who participated only at baseline (Table S2). Follow‐up participants were older and more likely to be female and married or living with a partner. Baseline GAD and STB were associated with lower odds of follow‐up participation, whereas baseline MDE was not significantly associated with attrition. A small but statistically significant difference in baseline general health status scores was also observed, while no significant differences were observed in baseline mental health status.

These findings suggest some potential for attrition bias; however, post‐stratification and inverse probability‐of‐censoring weights were applied to mitigate differential non‐response and attrition.

At baseline, the weighted prevalence of mental health conditions in this subsample was 6.1% for major depressive episode (MDE), 9.5% for GAD, and 12.4% for STB. At follow‐up, the prevalence of MDE increased to 8.8% and GAD to 18.4%, whereas the prevalence of STB decreased to 7.1%.

Regarding socio‐economic conditions during the pandemic, 42.9% of participants reported no indicators of job precariousness. Approximately half of the sample exhibited between one and three precarious employment indicators, and 1.1% met all five criteria, indicating severe job precariousness. In terms of material deprivation, 73.5% of participants reported no deprivation indicators, while 25.3% experienced some degree of deprivation. Severe material deprivation, defined as the inability to afford at least three essential items, was observed in 1.2% of the sample.

The distribution of all variables assessed is shown in Table 1, which summarizes the characteristics of the 922 participants across baseline and follow‐up measures.

### 3.2 Associations Between Pre‐Pandemic Mental Health and Socio‐Economic Outcomes

In bivariate analyses, baseline MDE and STB were significantly associated with job precariousness at follow‐up. Participants with MDE prior to the pandemic had higher odds of experiencing job precariousness during the pandemic (OR = 2.68, 95% CI [1.44, 4.98]), as did those with baseline STB (OR = 2.74, 95% CI [1.81, 4.14]). GAD at baseline was not significantly associated with job precariousness in bivariate models.

After adjustment for socio‐demographic characteristics, baseline employment status, physical comorbidities, disability, and body mass index, baseline GAD and STB remained independently associated with job precariousness. Individuals with pre‐pandemic GAD showed more than a threefold increase in the likelihood of job precariousness (adjusted OR = 3.24, 95% CI [1.15, 9.14]), and those with baseline STB also exhibited elevated odds (adjusted OR = 3.01, 95% CI [1.55, 5.84]). In contrast, the association between baseline MDE and job precariousness was no longer statistically significant after adjustment.

Baseline MDE, GAD, and STB were associated with material deprivation in bivariate analyses. However, these associations did not remain statistically significant in multivariate models adjusted for potential confounders, and no independent association was observed between baseline GAD and material deprivation. All results are shown in Table 2.

### 3.3 Associations Between Job Precariousness, Material Deprivation, and Changes in Mental Health Symptoms

Changes in mental health symptoms from baseline to follow‐up were examined in relation to exposure to job precariousness and material deprivation during the pandemic. Because job precariousness and material deprivation were assessed at follow‐up (T1) only, these models relate T1 exposure status to symptom change (T1–T0) and do not capture pre‐pandemic levels or within‐person changes in socioeconomic exposure.

In bivariate linear regression models, job precariousness was associated with a reduction in GAD symptoms (*β* = −0.176, 95% CI [−0.334, −0.017]) and STB severity (*β* = −0.334, 95% CI [−0.581, −0.087]). No significant association was observed between job precariousness and changes in depressive symptoms at this stage.

In multivariate models adjusting for socio‐demographic and health‐related covariates, job precariousness was associated with a decrease in depressive symptoms (*β* = −0.598, 95% CI [−0.919, −0.277]) and anxiety symptoms (*β* = −0.355, 95% CI [−0.528, −0.183]). Job precariousness was also associated with a reduction in STB severity (*β* = −0.577, 95% CI [−0.882, −0.272]). However, these associations were attenuated and no longer statistically significant after inclusion of resilience interaction terms in the fully adjusted models.

In contrast, material deprivation was positively associated with an increase in depressive symptoms (*β* = 0.497, 95% CI [0.256, 0.738]). This association remained statistically significant after adjustment for confounding variables (*β* = 0.496, 95% CI [0.253, 0.740]). All results are shown in Table 3.

### 3.4 Age‐Stratified and Interaction Analysis

The full estimates from the age interaction models, including main effects and interaction terms for job precariousness and material deprivation across mental health outcomes, are presented in Table 4. For depressive symptoms, material deprivation was significantly associated with greater symptom worsening (*B* = 0.684, *p* < 0.001), and this association was modified by age, with a significant interaction observed among younger participants (18–29 years) (*B* = −0.934, *p* = 0.036). Similarly, for job precariousness, a significant interaction with younger age was observed (*p* = 0.017), indicating that the effect of precariousness on depressive symptoms differed across age groups.

**TABLE 4:** Interaction between age groups and socioeconomic factors on changes in mental health outcomes.

Outcome	Exposure	Variable	*B* (95% CI)	*p*
MDE	JP	Precariousness	0.223 (−0.164, 0.609)	0.258
		Age 18–29	1.816 (1.073, 2.559)	< 0.001
		Age ≥ 66	0.464 (0.040, 0.888)	0.032
		Precariousness × Age 18–29	−1.136 (−2.068, −0.205)	0.017
		Precariousness × Age ≥ 66	−0.559 (−2.933, 1.815)	0.644
	MD	Deprivation	0.684 (0.403, 0.965)	< 0.001
		Age 18–29	0.500 (0.007, 0.993)	0.047
		Age ≥ 66	0.193 (−0.117, 0.503)	0.223
		Deprivation × Age 18–29	−0.934 (−1.807, −0.061)	0.036
		Deprivation × Age ≥ 66	−0.637 (−1.279, 0.005)	0.052
GAD	JP	Precariousness	−0.054 (−0.295, 0.186)	0.656
		Age 18–29	0.574 (0.243, 0.906)	0.001
		Age ≥ 66	0.035 (−0.254, 0.324)	0.813
		Precariousness × Age 18–29	0.080 (−0.412, 0.573)	0.748
	MD	Deprivation	0.067 (−0.067, 0.201)	0.326
		Age 18–29	0.183 (−0.035, 0.401)	0.100
		Age ≥ 66	0.053 (−0.182, 0.288)	0.660
		Deprivation × Age 18–29	−0.147 (−0.625, 0.331)	0.548
		Deprivation × Age ≥ 66	−0.175 (−0.595, 0.245)	0.417
STB	JP	Precariousness	−0.317 (−0.693, 0.059)	0.098
		Age 18–29	0.178 (−0.545, 0.900)	0.629
		Age ≥ 66	0.063 (−0.349, 0.475)	0.764
		Precariousness × Age 18–29	0.174 (−0.731, 1.080)	0.706
		Precariousness × Age ≥ 66	0.364 (−1.944, 2.672)	0.757
	MD	Deprivation	0.203 (−0.089, 0.495)	0.172
		Age 18–29	0.610 (0.090, 1.130)	0.020
		Age ≥ 66	0.411 (0.089, 0.733)	0.013
		Deprivation × Age 18–29	−0.862 (−1.772, 0.048)	0.063
		Deprivation × Age ≥ 66	−0.156 (−0.825, 0.513)	0.646

*Note:* Reference category for age = 30–65 years. Models estimated using inverse probability‐of‐censoring weights (IPCW) and post‐stratification weights.

Abbreviations: 95% CI = 95% confidence interval, GAD = generalized anxiety disorder, JP = job precariousness, MD = material deprivation, MDE = major depression episode, STB = suicidal thoughts and behavior.

For anxiety symptoms, neither job precariousness nor material deprivation showed significant associations, and no interaction effects with age were observed. Although younger participants tended to show greater increases in anxiety symptoms, these differences were not modified by socioeconomic exposures.

For suicide‐related outcomes, job precariousness was not associated with changes in risk, and no interaction effects were detected. In contrast, the model including material deprivation was statistically significant (*p* = 0.033), with age showing a significant effect: both younger (*p* = 0.020) and older participants (*p* = 0.013) exhibited greater increases in suicide‐related outcomes. The interaction between material deprivation and younger age approached statistical significance (*p* = 0.063), suggesting a potential age‐related difference that should be interpreted with caution.

Overall, these findings indicate that the role of age as a modifier varies depending on both the type of socioeconomic exposure and the mental health outcome considered.

### 3.5 Moderating Role of Resilience

Resilience was inversely associated with changes in depressive symptoms among individuals experiencing material deprivation. In models including interaction terms, higher resilience scores were associated with a reduction in the magnitude of depressive symptom increases linked to material deprivation (*β* = −0.075, 95% CI [−0.107, −0.042]). This moderating effect remained significant after full adjustment for confounders (*β* = −0.078, 95% CI [−0.111, −0.046]).

No significant moderating effect of resilience was observed in the associations between job precariousness and changes in anxiety symptoms or STB severity in fully adjusted models.

## 4 Discussion

This study yielded three main findings. First, GAD and STB present before the pandemic were independently associated with an increased likelihood of experiencing job precariousness during the COVID‐19 pandemic. Second, job precariousness was unexpectedly associated with reductions in depressive, anxiety, and suicide‐related symptoms in the first year. Third, material deprivation was associated with worsening depressive symptoms, and this association was attenuated among individuals with higher resilience. Across outcomes, we also observed outcome‐specific age‐related heterogeneity: age moderated associations for depressive symptom change (including significant interactions for younger adults), whereas no age‐related effect modification was evident for anxiety; for suicide‐related outcomes, age showed significant effects and the deprivation‐by‐age interaction suggested a possible trend in younger adults that warrants cautious interpretation.

### 4.1 Pre‐Pandemic Mental Health and Subsequent Job Precariousness

The finding that pre‐existing GAD and STB were associated with higher levels of reported job precariousness during the pandemic supports the notion that mental health vulnerabilities may precede socio‐economic difficulties. Individuals with anxiety or suicide‐related symptoms may face cumulative disadvantages in the labor market due to reduced work capacity, stigma, or difficulties maintaining stable employment under conditions of uncertainty. These results are consistent with prior research identifying precarious employment as both a determinant and a consequence of poor mental health (Benach et al. 2014; Vives et al. 2013).

Notably, major depressive episode (MDE) was not independently associated with job precariousness after adjustment for confounders. This may reflect heterogeneity in the functional impact of depressive episodes or differences in how depression, anxiety, and suicidal behaviors affect occupational functioning. Anxiety symptoms, in particular, may interfere more directly with job performance and adaptability during periods of rapid labor market change, while STB may capture a broader constellation of psychosocial vulnerability. It is also important to acknowledge that our measure of job precariousness incorporated subjective components, including fear of job loss or retaliation. Consequently, the observed associations may reflect not only greater exposure to precarious employment conditions but also differences in perceived employment insecurity among individuals with pre‐existing mental health conditions.

### 4.2 The Paradoxical Association Between Job Precariousness and Symptom Reduction

Contrary to expectations based on pre‐pandemic literature, job precariousness during the COVID‐19 pandemic was associated with reductions in depressive, anxiety, and suicide‐related symptoms. This finding contrasts with extensive evidence linking precarious employment to poorer mental health under non‐crisis conditions (Kim and Von Knesebeck 2015; Min et al. 2014; Stuckler et al. 2009). However, similar paradoxical patterns have been observed in other studies conducted during the pandemic, suggesting that the psychological meaning of employment instability may have shifted in this context.

A substantial body of COVID‐19 research has documented that financial strain, income loss, and job insecurity/economic hardship were associated with worse mental health outcomes, including higher depressive and anxiety symptoms, in population‐based samples. Longitudinal and repeated cross‐sectional evidence from the first pandemic year generally showed elevated psychological distress and a disproportionate burden among individuals experiencing economic disruption or reduced resources (Pierce et al. 2020). Against this background, our findings suggest that the short‐term mental health correlates of job precariousness, as operationalized here, may be phase‐ and context‐specific and may differ from the effects of more proximal constraints on basic needs.

One plausible explanation relates to the collective nature of the crisis. During the pandemic, job loss and employment instability affected entire sectors and social groups, potentially reducing individual perceptions of personal failure, shame, or social comparison. In such circumstances, precarious employment may have been reframed as a shared external stressor rather than an individual shortcoming, thereby attenuating its negative psychological impact. This interpretation is consistent with the possibility that, during a collective crisis, employment instability may be perceived as a shared external stressor rather than an individual shortcoming, thereby reducing processes of self‐blame and negative social comparison.

Several alternative explanations should also be considered. Individuals experiencing job precariousness may have benefited from temporary protective measures implemented during the pandemic, such as furlough schemes, income support, or enhanced social cohesion. Additionally, reduced work demands or relief from high‐pressure occupational environments may have contributed to short‐term symptom improvement for some individuals. Importantly, the attenuation of associations after inclusion of resilience interaction terms suggests that individual coping resources play a role in shaping these outcomes.

### 4.3 Associations Between Socioeconomic Adversity and Changes in Mental Health Symptoms and the Role of Age

Our interaction models suggest that the role of age is outcome‐specific when examining the mental health correlates of socioeconomic adversity. For depressive symptoms, material deprivation emerged as the most consistent risk factor, and the age‐by‐deprivation interactions indicate that the deprivation–depression link is not uniform across the life course, with younger adults showing a distinct pattern relative to the 30–65 reference group. A similar age‐related heterogeneity was observed for job precariousness in relation to depressive change, despite the absence of a robust overall main effect, supporting the interpretation that the mental health meaning of precarious employment may be particularly context‐dependent in younger populations. In contrast, for anxiety symptoms, neither job precariousness nor material deprivation showed clear associations or evidence of age‐related effect modification; rather, age differences appeared as a general pattern independent of these exposures. For suicide‐related outcomes, socioeconomic exposures were not directly associated with change and no interactions were detected, while both younger and older participants exhibited greater worsening compared with midlife adults, consistent with the multifactorial nature of suicide‐related phenomena and the likelihood that broader contextual, clinical, and temporal factors are needed for socioeconomic exposures to translate into detectable change. Methodologically, these findings reinforce the value of interaction models to capture age‐related heterogeneity that may be missed in stratified analyses, particularly when subgroup sizes are limited. Overall, the findings underscore the need to consider both age and the specific type of mental health outcome when evaluating the impact of socioeconomic adversity.

### 4.4 Material Deprivation and Depressive Symptoms

In contrast to job precariousness, material deprivation was consistently associated with increases in depressive symptoms. This finding aligns with a robust body of literature identifying financial strain and inability to meet basic needs as potent risk factors for depression, suicide‐related outcomes (Stuckler et al. 2009) and related mental health outcomes, including during the COVID‐19 pandemic (Ettman et al. 2020; Witteveen and Velthorst 2020).

Unlike employment instability, material deprivation directly constrains daily living conditions and may generate chronic stressors that are less amenable to cognitive reframing or collective normalization.

The observed moderating effect of resilience underscores its protective role in the context of economic hardship. Individuals with higher resilience scores experienced a smaller increase in depressive symptoms when exposed to material deprivation, suggesting that adaptive coping capacities may buffer the psychological impact of sustained financial strain. These findings are consistent with previous research highlighting resilience as a key factor in mitigating the mental health consequences of adversity (Bonanno et al. 2011; Connor and Davidson 2003).

### 4.5 Implications for Suicide Prevention and Public Health

From a suicide prevention perspective, these findings highlight the importance of distinguishing between different forms of socio‐economic adversity. While job precariousness during a collective crisis may not uniformly increase suicide‐related risk and may even be associated with short‐term symptom reduction, material deprivation appears to represent a more persistent and harmful stressor. Interventions aimed at reducing suicide risk during socio‐economic crises should therefore prioritize policies that prevent severe material deprivation and ensure access to basic resources.

Furthermore, the association between pre‐pandemic mental health conditions and subsequent job precariousness underscores the need for early identification and support for individuals with anxiety and suicide‐related symptoms, particularly during periods of economic instability. Integrating mental health services with employment and social support systems may help disrupt the bidirectional cycle between mental illness and socio‐economic vulnerability.

### 4.6 Limitations and Future Directions

Several limitations should be considered when interpreting these findings. First, job precariousness and material deprivation were measured only at follow‐up; therefore, we could not establish their pre‐pandemic levels or model within‐person change in exposure trajectories. This limits causal inference and complicates the temporal interpretation of associations with changes in mental health outcomes.

Second, mental health outcomes and STB were assessed through structured self‐report measures rather than clinician‐administered interviews; accordingly, estimates should be interpreted as probable conditions and may be affected by reporting and recall biases, particularly for suicide‐related phenomena. In addition, STB was captured using selected CIDI items and an internally constructed severity score, which may not fully reflect the dimensional complexity of STBs.

Third, the study relied on an online cohort with attrition between baseline and follow‐up. Although inverse probability‐of‐censoring weights and post‐stratification were used to mitigate selection and attrition bias, our sensitivity analysis suggested differential follow‐up participation by baseline characteristics, including age, sex, civil status, and mental health burden (e.g., GAD and STB; Table S2). Consistent with previous panel‐based longitudinal studies, this pattern may affect generalizability, and residual bias may remain if dropout was related to unmeasured factors associated with both socio‐economic adversity and mental health outcomes. Although baseline MDE did not differ significantly between responders and non‐responders, baseline GAD and STB were associated with lower odds of follow‐up participation (Table S2); therefore, residual attrition bias cannot be fully excluded despite inverse probability‐of‐censoring weighting. In addition, although the underlying Kantar Access Panel is probability‐based and calibrated to census margins, participation in this specific study depended on invitation and response within the panel. As with other online panel‐based cohorts, this design may be subject to coverage limitations and self‐selection (e.g., differential internet access or panel membership). These weighting procedures help approximate population margins, but they may not fully eliminate bias if participation is associated with unmeasured determinants of both exposure and outcome. The reasons for attrition cannot be determined with certainty; however, high loss to follow‐up is common in online panel‐based longitudinal studies and may reflect panel fatigue, changes in contact availability, and competing demands during the pandemic period.

Fourth, some instruments were not administered to the full baseline sample (e.g., GAD‐7 was administered to all participants screening positive for depression and to a randomized subset of those screening negative), resulting in a reduced sample size for certain analyses and potential loss of precision. Accordingly, while the overall longitudinal cohort included 922 participants, analyses involving longitudinal GAD outcomes were based on the subset with GAD‐7 data at both assessments (*n* = 313), which may reduce the stability and precision of those estimates.

Fifth, the prevalence of severe material deprivation was low in this cohort during the first pandemic year, which may have limited statistical power to detect smaller effects or effect modification and may partly explain null findings for some associations. In addition, weighting procedures can increase variance when some strata are under‐represented at follow‐up (e.g., the youngest age group); therefore, subgroup estimates should be interpreted cautiously as they may be less stable and less precise.

Age and labor‐force status are also relevant for interpreting these findings. The cohort included a sizeable proportion of older adults and retirees, for whom employment precariousness is not directly applicable. We therefore operationalized job precariousness among labor‐force active participants, and conclusions regarding precarious employment should be interpreted primarily in relation to working‐age/labor‐market–attached groups. By contrast, material deprivation can affect individuals across the life course, including retired adults, and may capture distinct vulnerability processes that are not reducible to labor‐market exposures.

Finally, as in all observational studies, residual confounding cannot be ruled out. We adjusted for a broad set of socio‐demographic and health‐related covariates, but we lacked granular information on occupation‐specific exposures, income trajectories, informal support, and the timing/intensity of policy measures (e.g., furlough schemes) that could influence both exposure to precariousness/deprivation and mental health changes. In addition, we did not capture individual furlough/ERTE or leave‐of‐absence status, which may have influenced both exposure to employment adversity and mental health trajectories during the first pandemic year.

Future research should incorporate repeated measurements of employment conditions and material deprivation across multiple time points, ideally with richer labor‐market indicators and longitudinal modeling of exposure trajectories. Studies spanning later phases of the pandemic and post‐pandemic periods are also needed to determine whether the short‐term symptom reductions observed for job precariousness persist, reverse, or reflect time‐limited contextual factors.

## 5 Conclusions

This study provides longitudinal evidence that pre‐existing mental health conditions, particularly GAD and STB, were associated with a higher likelihood of reporting job precariousness during large‐scale socio‐economic crises. Conceptually, these findings suggest that mental health may be related not only to subsequent employment circumstances but also to how employment insecurity is perceived and reported during periods of labor‐market disruption.

A second key contribution is the observation that the mental health correlates of job precariousness during the first pandemic year differed from patterns typically described in non‐crisis settings. In this context, job precariousness was associated with short‐term reductions in depressive, anxiety, and suicide‐related symptoms, a finding that may reflect crisis‐specific processes (e.g., normalization of employment instability, collective framing, and temporary protective measures). Importantly, this result should not be interpreted as evidence that precarious employment is benign, but rather that its psychological meaning and short‐term correlates may vary across macro‐social contexts and policy environments.

In contrast, material deprivation emerged as a consistent and clinically relevant risk factor for worsening depressive symptoms, with resilience attenuating this association. This pattern aligns with the notion that inability to meet basic needs represents a more persistent and proximal stressor than employment instability per se, and it highlights resilience as a modifiable protective factor with potential value for intervention design.

### 5.1 Implications

From a suicide prevention and public health perspective, the results suggest three practical priorities during socio‐economic crises: (1) early identification and proactive support for individuals with pre‐existing anxiety and suicide‐related symptoms, who appear more likely to experience or perceive job precariousness during periods of socio‐economic disruption; (2) prevention and rapid alleviation of severe material deprivation through social protection policies that secure basic resources; and (3) strengthening individual and community resilience (e.g., through accessible psychosocial support and coping‐focused interventions) as a complement to structural measures. Integrating mental health services with employment and social support systems may be particularly important to interrupt bidirectional pathways between mental ill‐health and socio‐economic vulnerability.

### 5.2 Generalizability

These findings are most directly applicable to working‐age adults in settings comparable to Spain during the first year of COVID‐19, where labor‐market disruption co‐occurred with temporary protective measures and collective crisis framing. In this context, job precariousness may capture crisis‐specific employment dynamics whose short‐term mental health correlates differ from those observed under non‐crisis conditions. By contrast, material deprivation reflects a more proximal constraint on basic needs and may represent a more transferable marker of risk across contexts, although its prevalence and severity, and thus its population impact, depend on baseline living standards and the generosity/timing of social protection. Replication across later pandemic phases and in settings with different welfare and labor‐market regimes is warranted to determine whether (a) the short‐term symptom patterns associated with job precariousness persist, reverse, or are specific to acute crisis conditions, and (b) the deprivation–depressive symptom association and resilience buffering generalize to periods with different levels of material hardship.

### 5.3 Take‐Home Message

During large‐scale socio‐economic shocks, preventing or rapidly alleviating material deprivation and proactively supporting individuals with pre‐existing anxiety and suicide‐related symptoms, while strengthening resilience, may be central to reducing downstream mental health and suicide‐related burden. Reported job precariousness should also be monitored as potential markers of vulnerability, although their short‐term mental health correlates may be context‐dependent under acute crisis conditions.

## Author Contributions

**Isabel Feria‐Raposo:** formal analysis. **Sheila Lopez‐Romeo:** writing – original draft. **Silvia Recoder:** writing – review and editing. **Marc Casajuana‐Closas:** writing – review and editing. **Pere Castellvi:** writing – review and editing, conceptualization, investigation, funding acquisition, supervision, formal analysis. **Andrea Miranda‐Mendizabal:** methodology, writing – review and editing. **Carlos G. Forero:** writing – review and editing, resources, conceptualization, investigation, funding acquisition. **Susana Subira‐Alvarez:** writing – review and editing.

## Funding

This work was supported by the Instituto de Salud Carlos III (ISCII‐FEDER Exp: PI16/00165) and Observatorio Social La Caixa (Call LL20‐2).

## Ethics Statement

The study protocol was approved by the relevant institutional ethics committee (Reg. Number: MED‐2020‐02). All participants provided informed consent prior to participation. Data were anonymized before analysis, and the study was conducted in accordance with the ethical principles applicable to research involving human participants.

## Conflicts of Interest

The authors declare no conflicts of interest.

## Supporting information


**Table S1:** Item‐level criteria for job precariousness and material deprivation.
**Table S2:** Differences between responders and non‐responders.
**Methods S1**. Operational definitions and scoring.

## Data Availability

The data that support the findings of this study are available from the corresponding author upon reasonable request. Access to the data may be subject to ethical and data protection restrictions.
