# Supplementary material for: Factors Associated With Job Precariousness and Material Deprivation, and Their Association With Mental Health During the COVID‐19 Pandemic
Source: Suicide Life Threat Behav. 2026 Jul 28;56(4):e70132. doi: 10.1111/sltb.70132 (PMC13415803; doi:10.1111/sltb.70132)
Supplement: Supplementary file 1 — Table S1: Item‐level criteria for job precariousness and material deprivation. Table S2: Differences between responders and non‐responders. Methods S1. Operational definitions and scoring. [file SLTB-56-0-s001.docx]

**Supporting Information**

**Manuscript title:** Factors associated with job precariousness and material deprivation, and their association with mental health during the COVID-19 pandemic

**Contents:**Methods S1. Operational definitions and scoring
Table S1. Item-level criteria for job precariousness and material deprivation

Table S2. Differences between responders and non-responders

**Methods S1. Operational definitions and scoring**

**Mental health indicators (T0).** Screening instruments were used for mental health indicators. Major depressive episode (MDE) was defined as meeting five criteria in the CIDI and presenting significant functional impairment. Generalized anxiety disorder (GAD) was defined as a GAD-7 score of 10 or higher together with functional impairment. Suicidal thoughts and behaviours (STB) were defined as positive endorsement of any suicidal ideation, plan, or attempt.

**GAD-7 administration scheme.** At baseline, the GAD-7 was administered to all participants with positive depression screening using the CIDI instrument and to a randomized 40% of participants with negative depression screening; at follow-up, it was administered to the entire sample. Longitudinal analyses involving GAD symptoms were therefore restricted to participants with GAD-7 data at both assessments.

**Job precariousness (T1).** Job precariousness ranged from 0 to 5 criteria: (a) job stability, (b) incomes, (c) job rights, (d) job insecurity and fear to ask for a job improvement, and (e) irregular job conditions. Job precariousness was considered present if an individual met at least one of these five criteria.

**Material deprivation (T1).** Material deprivation ranged from 0 to 4 criteria: (a) not able to go on vacation once a year, (b) not able to eat meat or fish every two days, (c) not able to pay a EUR 750 unexpected expense, and (d) not able to warm home in winter. Material deprivation was considered present if the participant could not afford at least 3 of these 4 items.

**Change scores (T1-T0).** Change in the number of symptoms was coded as a continuous variable defined as the difference between the number of symptoms at T1 and the number of symptoms at T0 (T1 minus T0).

**Weights.** Percentages and regression models were weighted using post-stratification and inverse probability-of-censoring weights.

**Table S1. Item-level criteria for job precariousness and material deprivation**

| Construct | Items/criteria | Scoring | Binary definition used in analyses |
| --- | --- | --- | --- |
| Job precariousness (T1) | (a) Job stability; (b) incomes; (c) job rights; (d) job insecurity and fear to ask for a job improvement; (e) irregular job conditions | Count 0-5 | Exposed = 1 or more criterion |
| Material deprivation (T1) | (a) Not able to go on vacation once a year; (b) not able to eat meat or fish every two days; (c) not able to pay a EUR 750 unexpected expense; (d) not able to warm home in winter | Count 0-4 | Deprived = 3 or more items |

Note: Item wording corresponds to the survey indicators used at follow-up (T1).

**Table S2. Differences between responders and non-responders**

| **Variable** | | **Total**  **N (%)/Mean(SD)** | **Baseline**  **N (%)/Mean(SD)** | **Baseline & Follow-up**  **N (%)/Mean(SD)** | **OR (95% CI)/Mean difference (SE)** | ***p-value*** | |  |
| --- | --- | --- | --- | --- | --- | --- | --- | --- |
| Age | | 49.92 (15.71) | 47.05 (16.30) | 53.19 (14.34) | 6.14 (0.69) | <0.001 | |  |
| Gender  Male  Female | | 969 (48.5)  1029 (51.5) | 544 (51.13)  520 (48.87) | 425 (45.5)  509 (54.5) | 0.80 (0.67-0.95) | 0.013 | |  |
| Weight | | 72.95 (15.01) | 73.4 (15.13) | 72.43 (14.86) | 0.97 (0.67) | 0.148 | |  |
| Civil status  Married/Living with a couple  Single  Separated/Divorced  Widowed | | 1132(56.66)  606 (30.33)  157 (7.86)  103 (5.16) | 577 (54.23)  368 (34.59)  74 (6.95)  45 (4.23) | 555 (59.42)  238 (25.48)  83 (8.89)  58 (6.21) | 1  0.67 (0.55-0.82)  1.16 (0.83-1.63)  1.34 (0.90-2.01) | <0.001 | |  |
| Mental health condition  MDE  GAD  STB | | 129 (6.46)  113 (15.52)  297 (15.29) | 76 (7.14)  77 (18.83)  185 (18.01) | 53 (5.67)  36 (11.29)  112 (12.23) | 0.78 (0.54-1.12)  0.55 (0.36-0.84)  0.63 (0.50-0.82) | 0.20  <0.001  <0.001 | |  |
| Mental health status | | 2.43 (0.85) | 2.41 (0.87) | 2.45 (0.83) | 0.04 (0.03) | 0.28 | |  |
| General health status | | 2.92 (0.66) | 2.88 (0.7) | 2.97 (0.62) | 0.09 (0.03) | <0.001 | |  |
| Note: 95% CI= 95% Confidence Interval; Ref.=Reference; MDE= Major Depression Episode; GAD= Generalised Anxiety Disorder; STB= Suicidal Thoughts and behaviour  n= number of participants included in the logistic regression models, SD=Standard Deviation; SE= Standard Error | | | | | |  |  |  |
